# Reductions in Cerebral Blood Flow Can Be Provoked by Sitting in Severe Myalgic Encephalomyelitis/Chronic Fatigue Syndrome Patients

**DOI:** 10.3390/healthcare8040394

**Published:** 2020-10-11

**Authors:** C (Linda) MC van Campen, Peter C. Rowe, Frans C Visser

**Affiliations:** 1 Stichting CardioZorg, 2132 HN Hoofddorp, The Netherlands; fransvisser@stichtingcardiozorg.nl; 2 Department of Paediatrics, John Hopkins University School of Medicine, Baltimore, MD 21205, USA; prowe@jhmi.edu

**Keywords:** orthostatic intolerance, cerebral blood flow, sitting, myalgic encephalomyelitis, chronic fatigue syndrome, severe disease, ME/CFS

## Abstract

Introduction: In a large study with myalgic encephalomyelitis/chronic fatigue syndrome (ME/CFS) patients, we showed that 86% had symptoms of orthostatic intolerance in daily life and that 90% had an abnormal reduction in cerebral blood flow (CBF) during a standard tilt test. A standard head-up tilt test might not be tolerated by the most severely affected bed-ridden ME/CFS patients. Sitting upright is a milder orthostatic stress. The present study examined whether a sitting test, measuring cerebral blood flow by extracranial Doppler, would be sufficient to provoke abnormal reductions in cerebral blood flow in severe ME/CFS patients. Methods and results: 100 severe ME/CFS patients were studied, (88 females) and were compared with 15 healthy controls (HC) (13 females). CBF was measured first while seated for at least one hour, followed by a CBF measurement in the supine position. Fibromyalgia was present in 37 patients. Demographic data as well as supine heart rate and blood pressures were not different between ME/CFS patients and HC. Heart rate and blood pressure did not change significantly between supine and sitting both in patients and HC. Supine CBF was not different between patients and HC. In contrast, absolute CBF during sitting was lower in patients compared to HC: 474 (96) mL/min in patients and 627 (89) mL/min in HC; *p* < 0.0001. As a result, percent CBF reduction while seated was −24.5 (9.4)% in severe ME/CFS patients and −0.4 (1.2)% in HC (*p* < 0.0001). In the ten patients who had no orthostatic intolerance complaints in daily life, the CBF reduction was −2.7 (2.1)%, which was not significantly different from HC (*p* = 0.58). The remaining 90 patients with orthostatic intolerance complaints had a −26.9 (6.2)% CBF reduction. No difference in CBF parameters was found in patients with and without fibromyalgia. Patients with a previous diagnosis of postural orthostatic tachycardia syndrome (POTS) had a significantly larger CBF reduction compared with those without POTS: 28.8 (7.2)% vs. 22.3 (9.7)% (*p* = 0.0008). Conclusions: A sitting test in severe ME/CFS patients was sufficient to provoke a clinically and statistically significant mean CBF decline of 24.5%. Patients with a previous diagnosis of POTS had a larger CBF reduction while seated, compared to patients without POTS. The magnitude of these CBF reductions is similar to the results in less severely affected ME/CFS patients during head-up tilt, suggesting that a sitting test is adequate for the diagnosis of orthostatic intolerance in severely affected patients.

## 1. Introduction

Orthostatic intolerance is defined as a clinical condition in which symptoms worsen upon assuming and maintaining upright posture and are ameliorated (although not necessarily abolished) by recumbency [1]. Myalgic encephalomyelitis/chronic fatigue syndrome (ME/CFS) patients have a high prevalence of orthostatic intolerance [1,2]. We reported in a study of 429 ME/CFS patients that 86% had orthostatic intolerance symptoms during daily life [2]. Furthermore, during a 30-min head-up tilt table test, 90% had an abnormal cerebral blood flow (CBF) reduction as assessed by extracranial Doppler measurements. This abnormal CBF reduction was not only present in ME/CFS patients with postural orthostatic tachycardia syndrome (POTS) and delayed orthostatic hypotension, but also in patients with a normal heart rate and blood pressure response during tilt testing. The mean CBF reduction of 26% in the entire study population with ME/CFS was significantly different from the 7% reduction observed in healthy controls in response to the same orthostatic stress.

Extracranial Doppler measurements may take between 2 and 7 min, and in our recent study, 15 patients were excluded because they were unable to maintain the upright position during the acquisition period [2]. Moreover, patients can develop post-exertional malaise after conventional 60–90 degree head-up orthostatic stress testing [1]. Due to these two restrictions, conventional orthostatic testing may not be feasible in severe ME/CFS patients. Miwa et al. described in a study of 44 ME/CFS patients that during a 10 min active sitting, test 30 patients (69%) reported orthostatic intolerance [3]. Moreover, in that study, orthostatic intolerance symptoms while sitting were more prevalent in patients with increased functional impairment.

Assuming that severe ME/CFS patients cannot tolerate prolonged standing during tilt testing and also that they have orthostatic symptoms during sitting, the aim of the current study was to test the hypothesis that reduced cerebral blood flow was present in severe ME/CFS patients during sitting.

## 2. Materials and Methods

### 2.1. Patients and Healthy Controls

From June 2011 to January 2018, 801 patients with the suspicion of ME/CFS visited the outpatient clinic of the Stichting CardioZorg. All patients were evaluated by the same clinician (FVC). During the first visit, it was determined whether patients satisfied the criteria for CFS and ME, taking the exclusion criteria into account [4,5]. Furthermore, in case of ME/CFS, the severity of the disease was classified. Disease severity was scored according to the International Consensus Criteria (ICC), with severity scored as mild, moderate, severe, and very severe [4]. The clinician also ascertained the presence or absence of orthostatic intolerance symptoms in daily life like dizziness/light-headedness, prior syncope and prior near-syncope, nausea, etc., as well as triggering events like standing in a line. In the present study, we analyzed 100 severe ME/CFS patients who had cerebral blood flow measurements while seated. A sitting test was chosen over a standing test or tilt-table test because patients were not able to stand for more than several minutes and we wanted to avoid the prolonged post-exertional malaise that can occur after tilt table testing. Furthermore, we recorded whether patients had a previous diagnosis of postural orthostatic tachycardia syndrome (POTS) or fibromyalgia. If those with POTS were being treated with cardio-active medications, patients were instructed to stop those drugs for the 3 days before testing. Patients taking pain medications and selective serotonin reuptake inhibitors continued taking these medications as usual. For comparison, 15 healthy controls were studied. None used heart rate or blood pressure lowering medications. The study was carried out in accordance with the Declaration of Helsinki. All ME/CFS patients gave informed, written consent. The study was approved by the medical ethics committee of the Slotervaart Hospital, Amsterdam, the Netherlands (reference number P1736 and P1450).

### 2.2. Sitting Test

**Figure 1:**
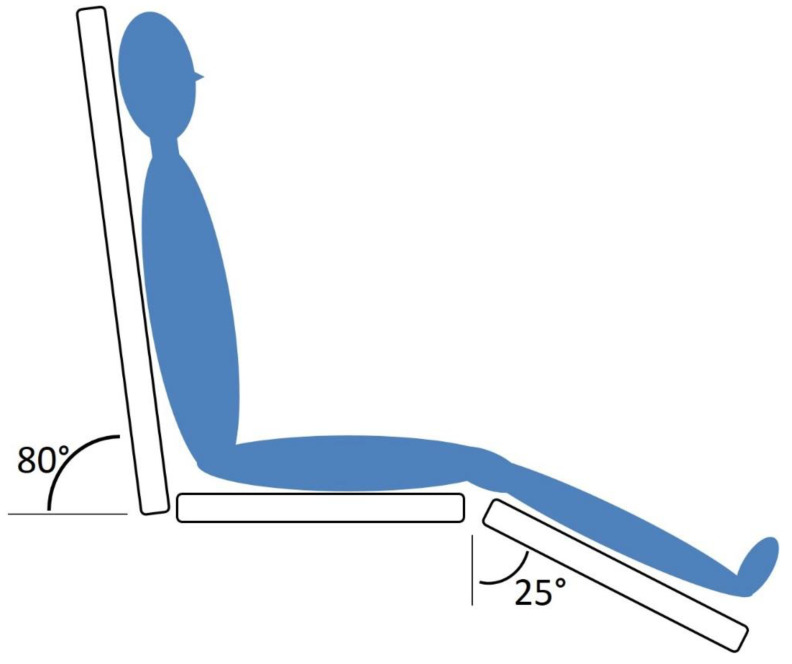
Research setup.

Testing was first performed in the seated position. Subjects had been seated for at least one hour before testing: see Table 1. Subjects were positioned on a special examination table with a whole trunk inclination of 80 degrees. The upper limbs rested comfortably at their sides and the lower limbs had an inclination of −25 degrees (see Figure 1). The study protocol started with measurements of heart rate and blood pressure, followed by extracranial Doppler echography (see below). After completion of the measurements, patients were placed in the supine position for 10–15 min, after which the measurements were repeated.

### 2.3. Cerebral Blood Flow Determination by Extracranial Doppler Echography

Cerebral blood flow measurements were performed with the same protocol as described in previous studies [2,6]. A more detailed description can be found in Appendix A. 

### 2.4. Statistical Analysis

Data were analyzed using Graphpad Prism version 8.2.4 (Graphpad software, La Jolla, CA, USA). All continuous data were tested for normal distribution using the D’Agostino & Pearson omnibus normality test, and presented as mean (SD) or as median with the IQR, where appropriate. Nominal data were compared with a Chi-square analysis. For continuous data, a paired and non-paired t-test/Mann–Whitney was used for comparison, where appropriate. Groups were compared using the ordinary one-way analysis of variance (ANOVA), with post hoc Tukey’s test in the event of a significant result. A *p* value of <0.01 was considered significant.

## 3. Results

### Patient Clinical and Echo Doppler Data

Between June 2011 and January 2018, 801 patients were evaluated. Of those, 53 patients did not fulfill the ME/CFS criteria, leaving 748 patients diagnosed with ME/CFS. Of the 748 ME/CFS patients, 177 (24%) were classified as having severe ME/CFS. From this group, 38 patients were excluded because they underwent a passive standing test, 12 patients were excluded from the analysis because of the use of blood pressure and/or heart rate influencing medication, 12 patients refused orthostatic stress testing, and 15 patients had incomplete or insufficient image acquisitions, leaving 100 patients with a severe grade of ME/CFS studied (88 females/12 males). The estimated mean duration of sitting before the start of the sitting test was 1 h 55 min (SD 58 min). The maximal duration of sitting before starting the test was 3.5 h. Baseline characteristics are presented in Table 2. For the patient group, median disease duration was 14 (IQR 8–22) years. Daily life orthostatic intolerance symptoms were reported by 90 of the 100 ME/CFS patients. At the time of the test, nine patients were being treated with selective serotonin reuptake inhibitors (SSRIs). Baseline and clinical data in patients with and without SSRIs did not differ significantly (data not shown). Fibromyalgia was present in 37/100 patients. Of the 37 ME/CFS patients with fibromyalgia, 28 were being treated with pain-medication or benzodiazepines for sleeping disorders. Of the remaining 63 patients, 4 were being treated with benzodiazepines for sleeping disorders. A diagnosis of POTS was reported by 34 patients. Table 2 also shows supine and sitting test results for both healthy controls and severe ME/CFS patients. No statistically significant differences were present during supine testing. Heart rate and blood pressure was similar between groups during the sitting position, but there was a significant difference in the cerebral blood flow during sitting: 474 (96) mL/min in ME/CFS patients vs. 627 (89) mL/min in healthy controls (*p* < 0.0001). The percent decline in cerebral blood flow in the sitting position compared to the supine position was −24.5 (9.4)% in ME/CFS patients vs. −0.4 (1.2)% in healthy controls (*p* < 0.0001). No patients developed POTS or delayed orthostatic hypotension during the test.

**Figure 2:**
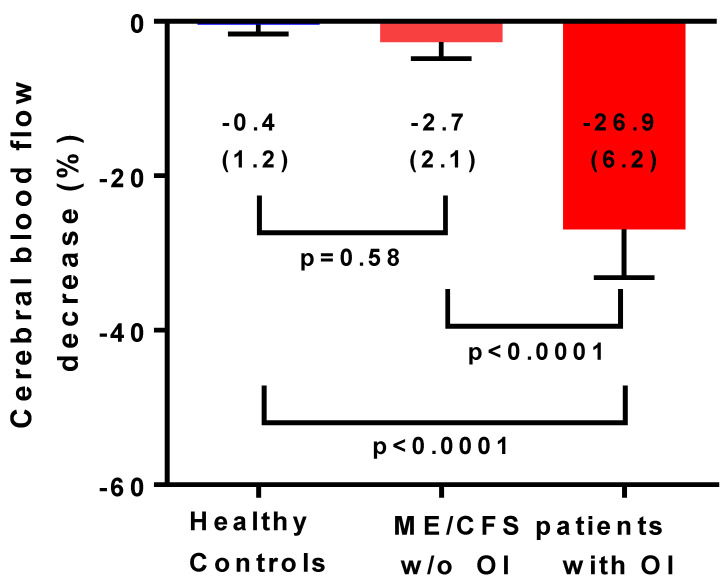
Cerebral blood flow decrease in percentage reduction between the supine position and the sitting position in healthy controls (*n* = 15), in severe ME/CFS patients without orthostatic intolerance in daily life (*n* = 10), and in severe ME/CFS patients with orthostatic intolerance in daily life (*n* = 90). OI: orthostatic intolerance; w/o: without; ME/CFS: myalgic encephalomyelitis/chronic fatigue syndrome; *p*-values shown are the result from an ordinary one-way analysis of variance (ANOVA), with post hoc Tukey’s test. Results are presented as percentage cerebral blood flow decrease (standard deviation).

Figure 2 shows the percent decrease in cerebral blood flow from supine to sitting in healthy controls and in severe ME/CFS patients with and without orthostatic intolerance in daily life. Only 10 (10%) severe ME/CFS patients had no orthostatic symptoms in daily life. Cerebral blood flow reductions in patients without daily life orthostatic intolerance symptoms did not differ significantly from those found in healthy controls.

Table 3 shows the baseline, supine, and sitting test results for severe ME/CFS patients with (*n* = 37) and without (*n* = 63) fibromyalgia. Patients with fibromyalgia were significantly older and had a higher BMI. Other variables did not differ significantly. The percent decline in cerebral blood flow was −23.9 (9.4)% and −24.9 (9.5)% for patients with and without fibromyalgia respectively (*p* = 0.51) Comparing these data in patients with and without pain-medication and/or benzodiazepines showed similar results (data not shown).

Table 4 shows the baseline and supine and sitting test results for severe ME/CFS patients with (*n* = 34) and without (*n* = 66) POTS in their history. Patients with POTS in their history were significantly younger. Other variables did not differ significantly. The percent decline in cerebral blood flow was −22.3 (9.7)% and −28.8 (7.2)% for patients without and with POTS in their history respectively (*p* < 0.001).

**Figure 3:**
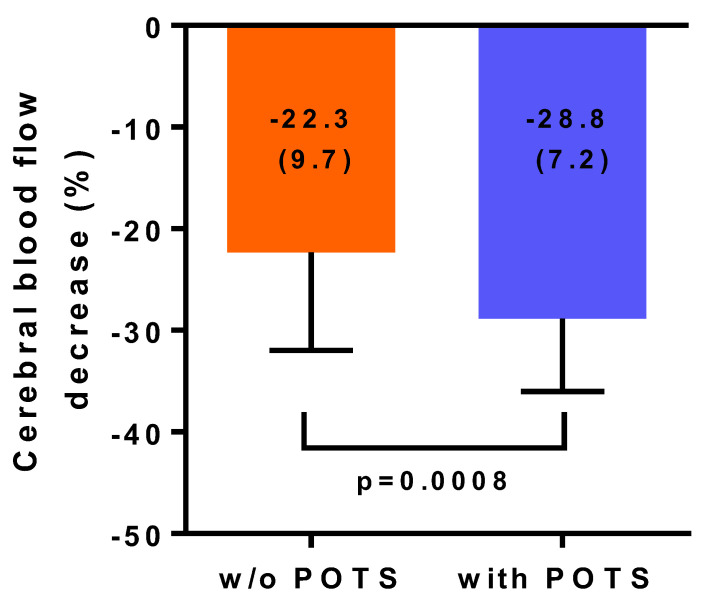
Cerebral blood flow reductions in percent reduction between the supine position and the sitting position in severe ME/CFS patients with (*n* = 34) and without (*n* = 66) a history of Postural Orthostatic Tachycardia Syndrome (POTS). A history of POTS could result from self-reporting, from the diagnosis with a standing test or a tilt-table test elsewhere in the past, or by tilt-table testing in our clinic in the past. POTS: postural orthostatic tachycardia syndrome; w/o: without. ME/CFS: myalgic encephalomyelitis/chronic fatigue syndrome; *p*-values shown are the result from an unpaired t-test. Results are presented as percentage cerebral blood flow decrease (standard deviation).

Figure 3 illustrates the percent reduction in cerebral blood flow between supine and sitting in severe ME/CFS patients based on the presence or absence of a history of POTS. Cerebral blood flow reductions in patients without POTS were significantly lower compared to those who had POTS (*p* = 0.0008).

## 4. Discussion

This is the first study showing that in severe ME/CFS patients, a significant reduction in cerebral blood flow (mean reduction −24.5%) can be provoked by sitting. In contrast, in healthy controls, only minimal change in cerebral blood flow reduction was observed (mean reduction −0.4%). Studying sitting as an orthostatic stressor with extracranial Doppler echography has not been described before. However, not all patients showed this abnormal response. In patients without daily life orthostatic intolerance complaints, a minimal reduction in cerebral blood flow of −2.7% was found, which did not differ significantly from healthy controls. In contrast, in patients with orthostatic intolerance complaints in daily life, sitting resulted in a 26.9% reduction in cerebral blood flow. This is similar to the 27% reduction in cerebral blood flow in an exploratory study with 20 degrees tilting during 15 min in severe ME/CFS patients [7]. It also is comparable to the 26% reduction observed after 30 min of 70 degree head-up tilt in a less severely affected population of ME/CFS patients [2]. We observed no difference in cerebral blood flow results whether patients had concomitant fibromyalgia or not. In patients with a previous diagnosis of POTS, cerebral blood flow reductions while seated were significantly larger than in patients who did not have POTS. It remains to be determined whether a sitting test would be adequate for the diagnosis of orthostatic intolerance/significant cerebral blood flow reduction in less severely affected ME/CFS patients. Furthermore, it remains to be determined whether a standard head-up tilt test shows more abnormal cerebral blood flow reduction than a sitting test in severe ME/CFS patients without orthostatic intolerance symptoms in daily life.

In the present study, a difference close to a significance of 0.05 was found between the sitting systolic blood pressure between healthy controls and severe ME/CFS patients: 122 (18) mmHg for healthy controls and 134 (18) mm Hg for ME/CFS patients. This difference might be explained by the fact that in severe ME/CFS patients, a decrease in blood volume may be present [8,9,10,11]. Moreover, we showed that cardiac output reductions during tilt-table testing were significantly more pronounced in ME/CFS patients compared to controls [12]. Both findings may result in a reduced venous return, and subsequent vasoconstriction and elevation of the measured, sitting, and systolic blood pressure. However, this remains to be proven. Also, it is unknown whether the duration of sitting before the test influences the reduction in venous return, cardiac output, and cerebral blood flow reduction in a ME/CFS patient population. In the present study, we did not find a correlation between the estimated duration of sitting before the start of the test and the percent reduction in cerebral blood flow (data not shown).

Miwa et al. described in a study of 44 ME/CFS patients (11 males and 33 females) that during an active standing test 40 (91%) reported orthostatic intolerance symptoms, and 30 (69%) reported orthostatic symptoms during a 10 min active sitting test [3]. Sitting as trigger for the development of orthostatic intolerance complaints was reported with increasing severity of the disease.

There are limited data on the effects of sitting using transcranial Doppler (TCD). Carter et al. studied the effects of 4-h sitting with and without exercise breaks in 10 healthy males [13]. Although they did not perform a statistical analysis of the difference of the change of 4 h sitting to supine afterwards, the data showed that middle cerebral artery velocity increased from 53.8 (1.6) cm/sec after 4 h of sitting to 55.5 (2.1) cm/sec in the supine position afterwards, without intervening exercise breaks. Their raw data imply that sitting middle cerebral artery velocity was 3% lower than supine middle cerebral artery velocity. Wheeler et al. demonstrated that prolonged sitting without exercise and/or breaks resulted in a significant reduction of the cerebral blood flow velocities from the middle cerebral arteries by transcranial Doppler over an 8 h period of sitting observation in 12 elderly obese subjects [14]. Perdomo et al. also observed a significant drop in middle cerebral artery velocities after 3 h and 40 min sitting in 25 subjects with pre-/stage 1 hypertension, with a return to baseline values at the end of the second working period [15]. No studies have reported transcranial Doppler measurements during sitting in ME/CFS patients. Unlike extracranial Doppler measurements, which calculate total CBF inflow, TCD can only measure CBF velocity, which is affected by vessel diameter, and in the presence of vasoconstriction can underestimate changes in CBF.

In the present study, a significantly higher reduction in cerebral blood flow was found in severe ME/CFS patients with POTS compared to severe ME/CFS patients without a history of POTS. The results are consistent with our previous study where POTS patients had a significant higher reduction in CBF at the end of 30 min of head-up tilt compared to patients with a normal heart rate and blood pressure response: −29 (6)% vs. −24 (10)%; *p* < 0.0005 [2]. Several previous studies have described a higher decline in cerebral blood flow as measured by TCD, but compared the subjects only to healthy controls and not to other ME/CFS patients with different hemodynamic tilt test results attributable to POTS [16,17]. Consistent with earlier studies, our ME/CFS patients with a history of POTS were younger than those without POTS [18,19]

### 4.1. Clinical Implications

Patients are advised to rest when they experience orthostatic intolerance complaints. Our findings of a clinically significant cerebral blood flow reduction while seated suggest that sitting may not be adequate enough to resolve symptoms of orthostatic intolerance in some patients. Importantly, none of the patients developed POTS or delayed orthostatic hypotension while sitting. This suggests that this form of stress testing may be less taxing than tilt-table testing or standing.

### 4.2. Limitations

This study only included severe ME/CFS patients. Sitting cerebral blood flow needs to be measured in less severely affected ME/CFS patients and compared to the blood flow measured in active standing and standard 70 degree angle tilt-testing. Whether differences in disease severity lead to differences in seated cerebral blood flow reduction needs to be studied in the future. While sitting was sufficient to provoke a clinically and statistically significant reduction in cerebral blood flow, we do not know whether it would have dropped further during head-up tilt. The measurement of supine cerebral blood flow was performed 10–15 min after patients had been sitting upright for at least an hour. We cannot exclude the possibility that there was a delayed recovery from the orthostatic stress, and that the supine measurement might have been different had patients not been sitting beforehand. However, supine measures did not differ between ME/CFS patients and controls, so we think that the supine measurements were not likely to have been affected by a carryover effect. Finally, while it is reasonable to expect that the sitting test would be less taxing than a standard tilt test, and therefore is less likely to provoke post-exertional malaise, this hypothesis remains to be tested.

## 5. Conclusions

This study demonstrates that a sitting test can result in a clinically significant reduction in cerebral blood flow in patients with severe ME/CFS and that sitting, therefore, should be considered as an important orthostatic stressor for the severe ME/CFS patient group. This method of orthostatic testing has the potential to improve the assessment of the prevalence of orthostatic intolerance in severely affected ME/CFS patients who are reluctant or cannot tolerate a standard 70 degree tilt test. In this patient population, a milder orthostatic stress was able to confirm cerebral blood flow abnormalities in the absence of heart rate and blood pressure abnormalities.

## Figures and Tables

**Table 1:** Summary of the sitting test procedure.

A minimum of 1 h sitting was required (including travel, time spent in the waiting room, and history taking)
Transfer to the research table and start sitting test
Measurement of heart rate and blood pressure in sitting position as shown in Figure 1
Cerebral blood flow measurements in sitting position
Lying down for 10–15 min
Measurement of heart rate and blood pressuring in supine position
Cerebral blood flow measurements in supine position
End of the sitting test

**Table 2:** Demographic features and orthostatic stress test results in healthy controls and severe ME/CFS patients.

	HC (*n* = 15)	Severe ME/CFS (*n* = 100)	*p*-Value
Male/female	2/13	12/88	0.88
Age (years)	38 (15)	38 (12)	0.94
Height (cm)	172 (9)	174 (8)	0.44
Weight (kg)	68 (15)	73 (16)	0.27
BMI (kg/m^2^)	22.9 (3.4)	24.3 (5.5)	0.33
Heart rate supine (bpm)	70 (14)	75 (15)	0.34
Heart rate sitting (bpm)	75 (10)	87 (19)	0.08
Systolic blood pressure supine (mmHg)	119 (14)	124 (17)	0.40
Systolic blood pressure sitting (mmHg)	122 (18)	134 (18)	0.05
Diastolic blood pressure supine (mmHg)	75 (12)	80 (11)	0.20
Diastolic blood pressure sitting (mmHg)	83 (10)	88 (11)	0.22
Cerebral blood flow supine (mL/min)	630 (85)	631 (121)	0.96
Cerebral blood flow sitting (mL/min)	627 (89)	474 (96)	<0.0001
Cerebral blood flow % change sitting vs. supine	−0.4 (1.2)	−24.5 (9.4)	<0.0001

HC: healthy controls; ME/CFS: myalgic encephalomyelitis/chronic fatigue syndrome.

**Table 3:** Baseline and orthostatic stress test results in severe ME/CFS patients with fibromyalgia (*n* = 37) and in severe ME/CFS patients without fibromyalgia (*n* = 63).

	FM Plus (*n* = 37)	FM Minus (*n* = 63)	*p*-Value
Male/female	2/35	10/53	0.12
Age (years)	43 (13)	36 (11)	<0.01
Disease duration (years) ^#^	15 (8.5–23.5)	13 (8–19)	0.46
Height (cm)	172 (7)	174 (8)	0.15
Weight (kg)	78 (17)	70 (15)	0.02
BMI (kg/m^2^)	26.3 (5.5)	23.1 (5.2)	<0.01
Heart rate supine (bpm)	71 (10)	78 (17)	0.02
Heart rate sitting (bpm)	81 (12)	91 (21)	0.02
Systolic blood pressure supine (mmHg)	125 (19)	124 (16)	0.95
Systolic blood pressure sitting (mmHg)	138 (20)	132 (16)	0.15
Diastolic blood pressure supine (mmHg)	82 (11)	79 (11)	0.24
Diastolic blood pressure sitting (mmHg)	90 (12)	86 (10)	0.09
Cerebral blood flow supine (mL/min)	639 (131)	627 (116)	0.62
Cerebral blood flow sitting (mL/min)	481 (88)	470 (101)	0.56
Cerebral blood flow % change sitting vs. supine	−23.9 (9.4)	−24.9 (9.5)	0.60

FM: fibromyalgia; ^#^ Data with median (IQR). ME/CFS: myalgic encephalomyelitis/chronic fatigue syndrome.

**Table 4:** Demographic and orthostatic stress test results in severe ME/CFS patients with and without a history of POTS.

	With POTS (*n* = 34)	Without POTS (*n* = 66)	*p*-Value
Male/female	2/32	10/56	0.18
Age (years)	31 (9)	42 (12)	<0.0001
Disease duration (years) ^#^	13 (8–18.5)	14.5 (–23)	0.47
Orthostatic intolerance in daily life	34 (100%)	56 (85%)	0.02 ^#^
Height (cm)	174 (7)	174 (8)	0.98
Weight (kg)	74 (18)	72 (15)	0.63
BMI (kg/m^2^)	24.7 (6.3)	24.1 (5.1)	0.59
Heart rate supine (bpm)	77 (18)	74 (13)	0.30
Heart rate sitting (bpm)	89 (21)	86 (17)	0.48
Systolic blood pressure supine (mmHg)	126 (16)	124 (18)	0.67
Systolic blood pressure sitting (mmHg)	136 (15)	134 (19)	0.64
Diastolic blood pressure supine (mmHg)	82 (12)	79 (10)	0.30
Diastolic blood pressure sitting (mmHg)	89 (11)	87 (10)	0.64
Cerebral blood flow supine (mL/min)	656 (121)	619 (121)	0.15
Cerebral blood flow sitting (mL/min)	466 (96)	478 (96)	0.57
Cerebral blood flow % change sitting vs. supine	−28.8 (7.2)	−22.3 (9.7)	<0.001

POTS: postural orthostatic tachycardia syndrome; ^#^ Data with median (IQR). ME/CFS: myalgic encephalomyelitis/chronic fatigue syndrome. ^#^ Chi-square testing (2 × 2 table).

## Appendix A

Internal carotid artery and vertebral artery Doppler flow velocity frames were acquired by one operator, using a Vivid-I system (GE Healthcare, Hoevelaken, the Netherlands) equipped with a 6–13 MHz linear transducer. Flow data of the internal carotid artery on the right and on the left side were obtained ∼1.0–1.5 cm distal to the carotid bifurcation. The vertebral artery data were obtained at the C3–C5 level. Care was taken to ensure the insonation angle was less than 60 degrees, that the sample volume was positioned in the center of the vessel, and that it covered the width of the vessel. High resolution B mode images, color Doppler images, and the Doppler velocity spectrum (pulsed wave mode) were recorded in one frame. The order of imaging was fixed: left internal carotid artery, left vertebral artery, right internal carotid artery, and right vertebral artery. At least two consecutive series of six frames per artery were recorded.

Blood flows of the internal carotid and vertebral arteries were calculated offline by a second investigator who was unaware of the patient severity status. Vessel diameters were manually traced on B-mode images, from the intima to the opposite intima. The surface area was calculated as follows: the peak systolic and end diastolic diameters were measured, and the mean diameter was calculated as: mean diameter = (peak systolic diameter × 1/3) + (end diastolic diameter × 2/3) [20]. The surface area = π * (diameter/2)^2^. Blood flow in each vessel was calculated from the mean blood flow velocities times the vessel surface area and expressed in ml/min. Flow in the individual arteries was calculated in 3–6 cardiac cycles and the data were averaged. Total cerebral blood flow was calculated by adding the flow of the four arteries. We previously demonstrated that this methodology had good intra- and inter-observer variability [6].
